# Mimicking the physical cues of the ECM in angiogenic biomaterials

**DOI:** 10.1093/rb/rbz003

**Published:** 2019-02-27

**Authors:** Cody O Crosby, Janet Zoldan

**Affiliations:** Department of Biomedical Engineering, The University of Texas at Austin, Austin, TX, USA

**Keywords:** angiogenesis, biomaterial–cell interaction, materials signal, vascular

## Abstract

A functional microvascular system is imperative to build and maintain healthy tissue. Impaired microvasculature results in ischemia, thereby limiting the tissue’s intrinsic regeneration capacity. Therefore, the ability to regenerate microvascular networks is key to the development of effective cardiovascular therapies. To stimulate the formation of new microvasculature, researchers have focused on fabricating materials that mimic the angiogenic properties of the native extracellular matrix (ECM). Here, we will review biomaterials that seek to imitate the physical cues that are natively provided by the ECM to encourage the formation of microvasculature in engineered constructs and ischemic tissue in the body.

## Introduction

The creation of mature, functional blood vessels is a critical challenge in the field of tissue engineering and regenerative medicine [1]. Broadly, vascularization in the human body can occur via three distinct yet cooperative mechanisms: vasculogenesis, angiogenesis, and arteriogenesis [2]. Vasculogenesis is defined as the assembly of neovessels from a progenitor cell population and is usually associated with embryogenesis. Angiogenesis describes the outgrowth of blood vessels from an existing, mature blood vessel, while arteriogenesis is defined as the consolidation and subsequent remodeling of existing collateral vessels. For the past two decades, research has focused on mimicking these mechanisms to vascularize tissue constructs, regenerate ischemic tissue and build vasculature *in vitro* for disease modeling and drug screening. However, these efforts have encountered severe technical challenges. Early attempts to treat ischemia and other vascular diseases involved the direct injection of angiogenic growth factors and stem/progenitor cells. Vascular endothelial growth factor (VEGF), one of the most well-studied proteins in biomedical science [3], was examined at the turn of the millennium as a noninvasive alternative to initiate angiogenesis at the diseased site. Promisingly, animal models responded well to the administration of exogenous VEGF and adeno-VEGF [4–6]; however, phase III clinical trials have shown limited benefits with no statistically significant improvement in vascular recovery in human patients [7, 8]. This phenomenon is generally attributed to the growth factors’ poor half-life in plasma (∼33 min) and the failure of adenoviruses to adequately transfect human cells with the VEGF-encoding plasmid [9, 10]. Cell-based therapies also emerged in the late 1990s/early 2000s and gained further traction with the discovery of endothelial progenitor cells (EPCs) by Asahara *et al*. [11, 12]. Though EPCs were demonstrated to participate in vasculogenic processes in animal models [12, 13], transplanted EPCs suffered from poor initial homing to the site of newly forming blood vessels and exhibited limited long-term viability [14].

**Figure 1.**
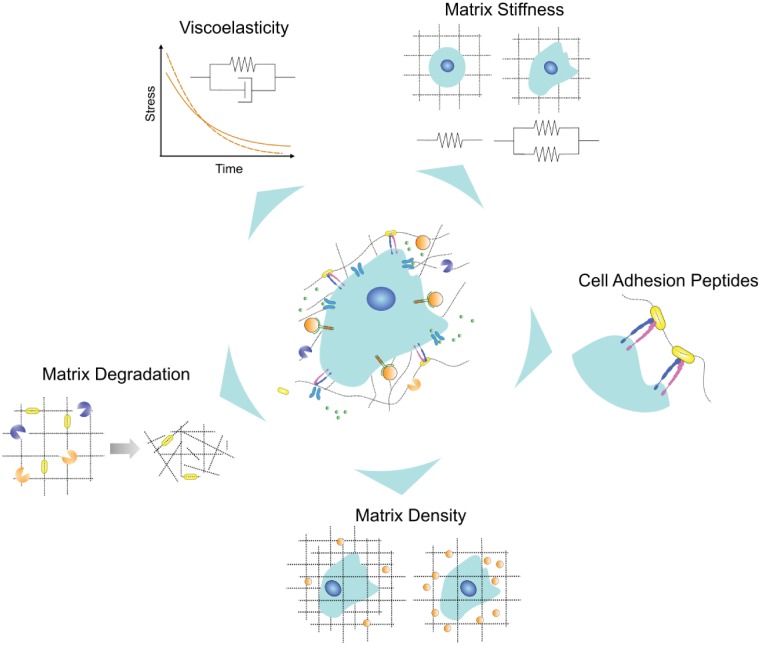
*ECs* in the native ECM respond to physical cues that are transduced through the architecture of fibrous structural proteins and receptor-binding peptides contained within the ECM. These physical cues should be incorporated into natural and synthetic matrices to create better angiogenic biomaterials and thereby maximize microvascular recovery

As novel angiogenic proteins and cell sources continue to be discovered, it has become clear that robust, nonimmunogenic materials must be developed to shield protein and cell cargo from the harsh ischemic environment. To develop these ‘angiogenic materials’, the following guidelines have been proposed: the material should (i) be biomimetic, (ii) deliver growth factors, (iii) display synergy between the material, cells and growth factors and (iv) be clinically relevant [15]. Angiogenic materials that promote the formation of healthy microvasculature are natural or synthetic polymers that retain water and are sensitive to the activity of encapsulated cells [16]. In other words, these materials are typically hydrogels that recapitulate critical components of the extracellular matrix (ECM), which, *in vivo*, provides vital physical and chemical cues to developing microvessels. Furthermore, endothelial and perivascular cells respond to mechanical forces transduced by the local ECM by activating signaling pathways that regulate cell viability, migration and morphology [17–19]. Here, we will review how angiogenic biomaterials have been designed to provide appropriate physical cues to stimulate microvascular recovery. We will highlight the critical role of ECM characteristics such as density, stiffness, viscoelasticity, degradation and presentation of integrin-binding motifs in the development of ECM-mimicking angiogenic biomaterials (Fig. 1).

## Matrix density

ECM-mimicking biomaterials primarily consist of an aggregation of fibrous, bioactive polymers. Generally, increasing the polymer concentration, or density, will enhance the structural integrity of the material. However, continually increasing the density of ECM fibers tends to impair the development of microvascular networks. Here, we will focus on this delicate tradeoff as we review how modulating ECM density guides the self-assembly of microvasculature.

**Figure 2.**
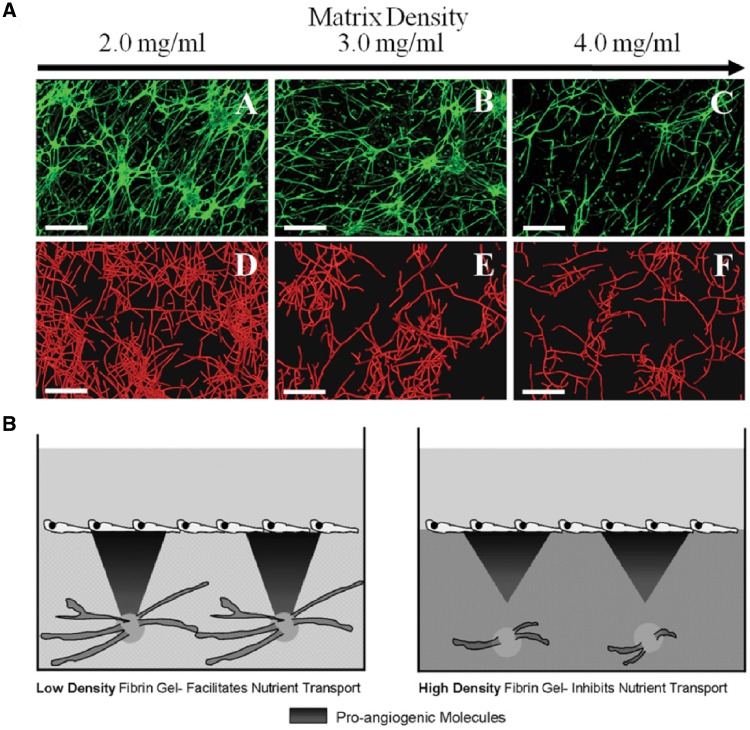
An intermediate concentration of ECM proteins is essential for robust microvascular regeneration. (A) Increasing collagen density abolished the growth of microvessels in both an experimental setup (microvessels are labeled with isolectin IB4-Alexa 488) and a computational model (microvessels are outlined in red). Reprinted from [26] with permission. (B) Increasing diffusivity encourages the transport of pro-angiogenic molecules, stimulating sprouting angiogenesis. Reprinted from [23] with permission from Cell Press

One of the first studies to systematically examine the effect of ECM density on microvasculature in biomaterials was conducted by Vernon and Sage [20]. They developed the radial invasion of matrix by aggregated cells (RIMACs) model to mimic endothelial cell (EC) migration and morphogenesis *in vitro*. RIMAC revealed that bovine aortic EC invasion distance decreased by 50% when the density of collagen hydrogel was increased by one order of magnitude; however, the shorter invasion distance encouraged the formation of low-volume, highly branched networks of capillary-like structures; meanwhile, ECs embedded in low-density hydrogels exhibited minimal branching. Similar results were also observed by others using a different model, the microcarrier-based assay, to mimic EC migration *in vitro* [21]. In this model, ECs were incubated with gelatin-coated Cytodex^®^ microcarriers to allow for cell attachment and were subsequently embedded in a fibrin hydrogel; fibroblasts were added to this system as a source of angiogenic growth factors. When fibroblasts were seeded on top of the hydrogel, the formation of capillary-like structures was hindered by increasing fibrin concentration; when fibroblasts were encapsulated along with the EC-microcarriers, vascular network formation was robust regardless of the density of the surrounding fibrin hydrogel [22–24]. As an alternative to sprouting angiogenesis assays, a microvessel three-dimensional (3D) model in collagen hydrogels was established in 1999 and remains in use today [25]. By combining this *in vitro* microvessel platform with a previously developed computational model, it was demonstrated that high-density collagen resulted in the development of shorter, less branched and more poorly connected microvessels [26] (Fig. 2A). It is possible that the positive correlation between matrix density and branching revealed by RIMAC may be attributed to the relative simplicity of the platform; for example, the other models described in this review add supporting cell types and more closely mimic physiological vasculogenic/angiogenic processes.

Nevertheless, these studies have revealed a clear trend: lower structural ECM protein concentrations allow ECs to proliferate and migrate more quickly in 3D microenvironments, thereby enabling the formation of vascular networks. Possible explanations for this widely observed phenomenon have been associated with changes in local ECM diffusivity (i.e. pore size), matrix anisotropy or protease activity, as further outlined in the following sections.

### Local diffusivity/pore size

When engineered matrices become denser, their pore size decreases; smaller pore sizes lengthen the diffusion time and limit the penetration depth of angiogenic growth factors. Experimental and computational studies have further illuminated the details of this mechanism. For example, an application of the Stokes–Einstein and Wilke–Chang relations to a fibrin model of sprouting angiogenesis confirmed that the diffusive transport of pro-angiogenic factors is limited in high ECM density hydrogels. Therefore, it was proposed that the relative diffusivity was partly responsible for the observed differences in sprouting from ECM-coated spheroids [23] (Fig. 2B). A random walk model that also accounted for cell heterogeneity and local matrix anisotropy confirmed experimental evidence that cells tend to diffuse faster and are more persistent in lower density collagen matrices [27]. However, the density of structural proteins cannot be simply minimized, as excessively low-density hydrogels do not provide adequate structural support for EC migration and attachment. Not surprisingly, intermediate collagen densities (1.2 and 1.7 mg/ml) were found to promote the greatest number of endothelial sprouts while a lower collagen concentration (0.7 mg/ml) lead to the development of unstable and broken sprouts [28, 29].

### Matrix anisotropy

It is hypothesized that the existence of an optimal matrix density is closely linked to the alignment (i.e. anisotropy) of the matrix fibers. As matrix density increases, the fibers are less likely to be randomly aligned (i.e. isotropic) near the sprouting vessel. Therefore, the sprouting tip cells that participate in angiogenesis are less likely to sense a local anisotropic disturbance, which is thought to induce local branching. This hypothesis has been explored in a computational model of sprouting angiogenesis; as the matrix density increased, the bulk fibers became homogeneous and thereby deprived the stimulated cells of crucial physical branching cues. This deprivation resulted in the formation of long, singular vessels [30].

### Proteolytic activity

The formation of stable endothelial sprouts relies on a delicate balance between stalk cell proliferation and sprouting tip cell migration [31]. It is hypothesized that EC-generated proteases are unable to sufficiently degrade high-density matrices, thus reducing the tip cell migration speed. This insufficient degradation destabilizes the elongating sprout because the equilibrium between the two synergistic angiogenic mechanisms is disrupted. To increase protease concentration and thereby increase the degradation of high-density fibrin hydrogels, Ghajar *et al*. co-cultured human mesenchymal stem cells (hMSCs) and ECs within fibrin hydrogels. The presence of hMSCs contributed to additional matrix metalloproteinase activity and increased sprouting from embedded EC-coated spheroids even in high-density fibrin hydrogels, demonstrating that increasing matrix density also requires a corresponding increase in proteolytic activity to promote adequate vessel formation [22].

The above-discussed findings were observed in biomaterials following *in vitro* experiments utilizing ECs extracted from mammalian tissue; similar trends in the effect of ECM density on the formation of microvasculature were also observed *in vivo* and with other sources of ECs. For example, Critser *et al*. [32] seeded endothelial colony forming cells (ECFCs) derived from human umbilical cord blood in collagen hydrogels of varying density and then implanted these constructs into immunodeficient mice, allowing neovessels to develop and anastomose with host vasculature. It was observed that three times as many blood vessels formed per normalized volume in the lowest density hydrogel (0.5 mg/ml) than the highest density hydrogels (3.5 mg/ml). Also, vessels in the lowest-density hydrogels exhibited the smallest cross-sectional area. While most studies have utilized human umbilical vein endothelial cells (HUVECs) or other primary mammalian ECs, Bezenah *et al*. [33] studied the response of induced pluripotent stem cell-derived ECs (iPSC-ECs) to varying matrix density in a microcarrier-based platform. Predictably, iPSC-ECs grew 25% fewer sprouts in the highest-density fibrin hydrogels (10 mg/ml) than the lowest density fibrin hydrogels (2.5 mg/ml); yet, when compared to HUVECs, iPSC-ECs displayed deficiencies in capillary morphogenesis.

In conclusion, ECM protein concentration must be carefully optimized to ensure that angiogenic biomaterials enhance vascularization while remaining functionally stable. Altering ECM matrix density also affects another critical material parameter: stiffness.

## Matrix stiffness

The stiffness of the ECM plays an instrumental role in regulating EC proliferation, signaling and differentiation (as reviewed by [34, 35]). For example, substrates of different stiffnesses can regulate the differentiation of murine bone marrow-derived EPCs toward a specific EC phenotype; specifically, very stiff substrates (10:1 PDMS, stiffness of ∼130 kPa) upregulated an arterial phenotype, while softer substrates (40:1 PDMS, stiffness of a ∼5 kPa) encouraged a venous phenotype [36]. Changes in substrate stiffness have been manifested in many pathological states and can contribute directly to vascular abnormalities. In breast cancer, the overexpression of lysyl oxidase by embedded cancer cells increased local ECM stiffness (by increasing the number of collagen crosslinks); this, in turn, promoted tumor cell invasion [37]. Furthermore, the matricellular protein CNN1/CYR61, which is upregulated by ECs in response to increased stiffness, is responsible for the upregulation of N-cadherin in ECs; increased expression of N-cadherin allows metastatic cells to bind to the endothelium and begin the process of extravasation [38]. Despite these illuminating advances, a definite correlation between stiffness and the regeneration of microvasculature remains challenging to elucidate in 3D models.

**Figure 3.**
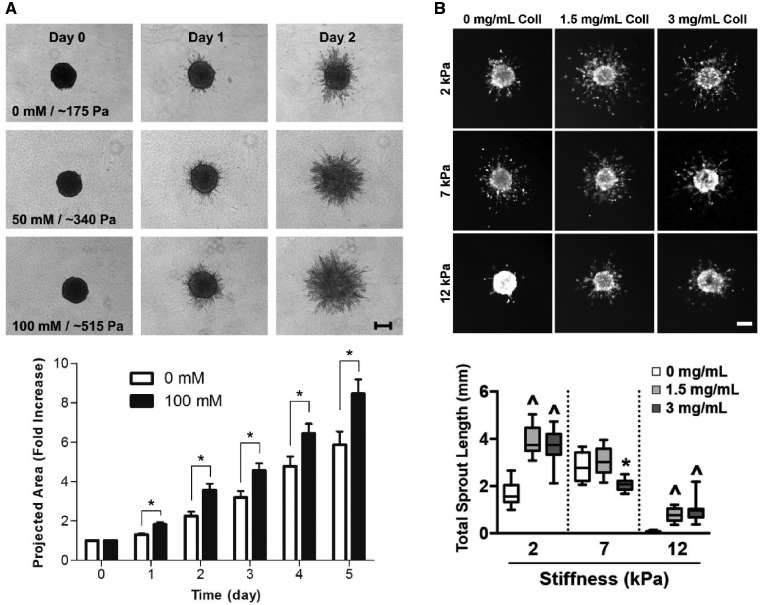
Modeling *in vitro* sprouting angiogenesis using EC-coated spheroids embedded in hydrogels of varying stiffness reveals conflicting behavior. (A) ECs in glycated collagen hydrogels show increased sprouting and maintain a larger projected area in stiffer hydrogels. (B) ECs in calcium-crosslinked collagen/alginate hybrid hydrogels show a decreased angiogenic response in stiffer hydrogels; this trend holds irrespective of ECM density. Reprinted from [42] and [45], respectively, with permission from Elsevier

It is challenging to isolate separate changes in stiffness from changes in local density. While increasing ECM density increases stiffness, it also decreases the pore size (i.e. diffusivity), increases the number of bioactive ligands available to encapsulated cells and alters local fiber architecture [27]. For example, a novel self-assembling protein hydrogel was designed to permit enhanced control over hydrogel stiffness and the number of integrin-binding sites by varying the concentration of adhesive peptides, (acetyl)-GRGDSP-GG-FKFEFKFE-CONH2 (KFE-RGD) and (acetyl)-FKFEFKFE-CONH2 (KFE) [39]; however, in this system it is not clear that the observed differences in bulk elastic modulus can be separated from varying protein density. Several groups have developed new means to crosslink collagen hydrogels that can overcome this inherent limitation. For example, ribose was employed to nonenzymatically glycate type I collagen hydrogels; by varying the concentration of ribose from 0 to 250 mM, collagen hydrogels with increasing stiffness from ∼100–200 Pa to 1 kPa were generated without modulating the density of collagen within the hydrogel [40, 41]. Increasing the stiffness of these collagen hydrogels led to increased EC spreading and increased sprouting from EC-coated spheroids [42] (Fig. 3A). Microbial transglutaminase (mTG), an industrial protein additive, was used to enzymatically crosslink collagen without affecting matrix density, pore size or overall porosity [43]. mTG-treated collagen hydrogels better stimulated angiogenic sprouting of EC monolayers compared to collagen hydrogels incubated with lower concentrations of mTG (i.e. lower stiffness hydrogels). Alternatively, the stiffness of collagen hydrogels was varied independently of matrix density by adding poly(ethylene glycol) di-(succinic acid *N*-hydroxysuccinimidyl ester), PEG-diNHS, thereby increasing the number of covalent bonds between the primary amines on adjacent collagen fibrils; however, this system was only used to encapsulate cancer cells, and the response of ECs remains untested [44]. Berger *et al*. [45] designed a novel interpenetrating network (IPN) of gelatin-methacrylate (GelMA) and collagen to independently tune hydrogel stiffness and density; increasing collagen concentration increased the density of the fibrous network while increasing GelMA acrylation increased the stiffness of the network upon exposure to ultraviolet (UV) radiation (Fig. 3B). Notably, it was observed that an increased density of collagen in the hybrid hydrogel encouraged angiogenic sprouting from EC-coated spheroids embedded in the hydrogel while decreasing the stiffness independently of the density reduced the total area of the endothelial sprouts. Most studies that have successfully decoupled matrix density from bulk material stiffness directly modulate the number of crosslinks in the engineered matrix by regulating the number of binding sites or by changing the concentration of naturally crosslinked materials. It is important to note that a change in crosslinker density affects the mesh size of the matrix and could, therefore, alter local diffusivity.

Though advances have been made to decouple density from stiffness, existing literature describing the impact of stiffness on EC morphology and the subsequent development of microvasculature in hydrogels is conflicting. This conflict becomes evident when network formation in 2D is compared to network formation in 3D microenvironments (Table 1).

**Table 1.** Effect of material stiffness on microvascular morphogenesis in 2D and 3D

Material	Dimensions	Effect of stiffness on EC behavior	Reference
Gelatin-polyacrylamide	2	HUVEC self-organize into extended structures on softer hydrogels	Deroanne *et al.* [46]
Collagen-polyacrylamide	2	HUVECs form single-cell networks on hydrogels with Young’s moduli of less than 1 kPa	Califano *et al.* [47]
PEGDA-GelMA	2	HUVECs develop more sprouts on 11–36 kPa hydrogels than 78 kPa hydrogels	Wu *et al*. [48]
RGD/GFOGER polyacrylamide	2	HUVECs form stable networks that are destabilized by growth factor addition on soft hydrogels (∼140 Pa). HUVECs do not self-assemble on stiff matrices, with or without growth factors	Saunders *et al.* [49]
Fibronectin/collagen-polyacrylamide	2	Different endothelial subtypes proliferate more when placed on a stiffer substrate; migration trends were specific to endothelial subtype	Wood *et al.* [50]
Methacrylated hyaluronic acid (MeHA)	2	Stiff matrices disrupt cell-to-cell junctions and increase the size of focal adhesions	Lampi *et al.* [51]
mTG-crosslinked collagen	2/3	HUVECs invade greater distances in stiffer mTG-crosslinked matrices; lumen diameters were similar across mTG concentrations	Lee *et al.* [43]
pH-adjusted collagen	2/3	Bovine pulmonary microvascular endothelial cells form thin, dense networks in stiffer collagen hydrogels; networks are more extensive and penetrate deeper into stiffer hydrogels	Yamamura *et al.* [52]
Glycated collagen	3	Stiffer hydrogels promote the formation of larger, more tortuous networks. Network size difference loses significance with extended culture	Francis-Sedlak *et al.* [53]
Glycated collagen	3	Stiffer hydrogels increase the number and length of sprouts from EC-coated spheroids in culture	Mason *et al*. [42]
Fibrin	3	ECs formed fewer and shorter extensions in hydrogels with a high degree of crosslinking	Urech *et al.* [54]
Alginate/GelMA	3	Stiffer hydrogels reduce the number and length of sprouts from EC-coated spheroids in culture	Berger *et al.* [45]
PEG-PQ (+/– Alloc)	3	Softer hydrogels promote greater neovascularization in murine models	Schweller *et al.* [55]
GelMA/LAP	3	Stiffer hydrogels (i.e. with a higher concentration of photoinitiator) discourage network formation; hydrogels with more cells have a higher branching index	Monteiro *et al.* [56]

Decreasing the stiffness of 2D materials promoted network assembly from ECs; however, this trend mostly disappeared when ECs were cultured in 3D microenvironments. There are several explanations for this phenomenon. First, we observed that the range of elastic moduli employed by these studies differed significantly. For example, in the study conducted by Mason *et al*., the glycated collagen hydrogels maintained elastic moduli on the order of a few hundred Pascals; in contrast, the hydrogels fabricated by Berger *et al*. exhibited elastic moduli on the order of thousands of Pascals. Therefore, it is possible that ECs exhibit a Gaussian-like response to 3D stiffness, i.e. low stiffness values destabilize the networks and high stiffness values limit the expansion of neovessels. Second, we note that a definite trend that only appears in 2D culture could be misleading. For decades, it has been recognized that 3D culture is a more faithful mimic of physiological conditions than stiff polystyrene plates or thin, pseudo-3D materials, e.g. electrospun scaffolds. In the body, cells are constrained in three dimensions; in response, cells alter their geometry, proliferation and secretions to the surrounding ECM, i.e. they significantly remodel their surrounding environment [57]. Third, it has been suggested that the nebulous effect of stiffness on ECs cultured in 3D microenvironments may be attributed to a failure to quantify stiffness on an appropriate length-scale. For example, it was determined that the tips of sprouting capillaries tended to be colocalized with regions of highly variable stiffness as measured by optical tweezers-based microrheology [58]. Though these hydrogels maintained a relatively soft bulk modulus for the duration of the 2-week experiment, bulk rheology was unable to capture the dynamic ECM remodeling that is a critical mediator of capillary morphogenesis. Recently, it has become apparent that changes in bulk stiffness may be masking variability in another crucial material parameter: viscoelasticity.

## Viscoelasticity

Recently, it has become evident that studying the elastic modulus in isolation does not capture the complex mechanical signaling that may be imparted by angiogenic biomaterials [59, 60]. As cells migrate through a biological matrix, they attach to the ECM and exert force on the ECM fibrils. However, the stiffness of the surrounding matrix rapidly fluctuates as this exerted force is dissipated by cells remodeling the surrounding ECM. Therefore, to be considered ECM-mimicking materials and thus faithful mimics of the angiogenic microenvironment, angiogenic biomaterials should be able to dissipate stress and exhibit a time-dependent mechanical response, i.e. demonstrate viscoelastic properties. However, the impact of matrix viscoelasticity on encapsulated cells is relatively unexplored and has been limited to the study of hMSCs or murine myoblasts.

Furthermore, distinguishing the effect of matrix viscoelasticity from the effect of matrix elastic properties on cell behavior is a significant challenge in many existing biomaterials. Many synthetic, engineered matrices are crosslinked with covalent bonds, which creates materials with nearly pure elastic behavior; if the synthetic materials display viscoelastic properties, they are invariably coupled to the bulk elastic modulus of the material. In contrast, most common ECM-derived proteins currently used in tissue engineering already demonstrate optimized viscoelastic properties [61].

Several groups have developed different strategies to decouple the stiffness of engineered matrices from their viscoelastic properties. Cameron *et al*. [62] tailored the concentrations of *Acryl* and *Bis* in polyacrylamide hydrogels to allow the bulk loss modulus to be varied independently of the bulk compressive (elastic) modulus. hMSCs grown on hydrogels with a loss modulus of about 130 Pa proliferated more, displayed immature (i.e. less than 3.5 μm) focal adhesion formation and maintained elevated smooth muscle gene expression (∼40%) compared to hMSCs cultured on hydrogels with lower loss moduli. Subsequent studies provided a molecular-scale mechanism, linking elevated smooth muscle gene expression with Rac Family Small GTPase 1 (Rac1) activation in hMSCs cultured on high loss moduli hydrogels [63].

**Figure 4.**
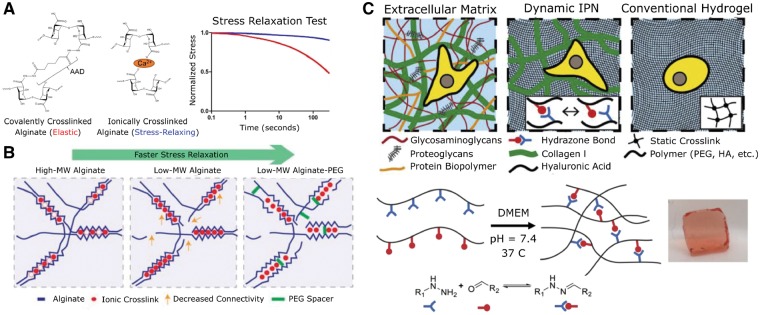
Materials with viscoelastic properties better recapitulate the mechanical milieu of the native ECM. (A) Alginate crosslinked covalently with adipic acid dihydrazide remained mostly elastic. In contrast, alginate crosslinked via calcium dissipated more than half of the absorbed stress within minutes. Reprinted from [66] with permission from Elsevier. (B) By introducing lower molecular weight alginate and PEG spacers, Chaudhuri *et al*. were able to create biomaterials that more closely mimicked the natural viscoelastic properties of the ECM. Reprinted from [67] with permission from Nature Publishing Group. (C) An IPN of collagen and hydrazone-bonded hyaluronic acid promoted cell spreading and fiber remodeling. Reprinted from [69] with permission from Elsevier

Crosslinking alginate with the sequential addition of hydroxybenzotriazole, 1-ethyl-3-(3-dimethylaminopropyl) carbodiimide (EDC) and adipic acid dihydrazide led to the formation of strong covalent bonds between carboxylic groups and generated alginate hydrogels that did not readily dissipate stress under an applied load. In contrast, ionic bonds formed by calcium-mediated crosslinking of G blocks on different alginate chains readily dispersed stress and led to plastic deformation of the hydrogel under an applied load [64] (Fig. 4A). These two methods served as a foundation to evaluate the effect of stress relaxation on the spreading, proliferation and yes-associated protein (YAP) nuclear translocation of hMSCs [65]. While hMSCs typically do not spread or form focal adhesions on low elastic moduli substrates, hMSCs spread considerably and formed strong focal adhesions even on substrates with demonstrably low elastic moduli (1–2 kPa), thereby implying an essential role for viscoelasticity in hMSC mechanotransduction. Mouse myoblasts seeded on these alginate hydrogels demonstrated similar behavior; myoblasts grown on ionically crosslinked alginate hydrogels became more extended and proliferated at a higher rate compared to myoblasts cultured on EDC-crosslinked alginate [66]. Varying the molecular weight of the alginate and introducing poly(ethylene) glycol (PEG) spacers enabled the generation of ionically crosslinked hydrogels with varying viscoelastic properties [67] and further validated the previously observed interrelation between cellular behavior and stress-relaxing substrates (Fig. 4B) [65, 66]. Therefore, modulating the viscoelastic properties of hydrogels could be a powerful new tool to control stem cell fate and has the potential to regulate the vasculogenic potential of ECs or EPCs.

In addition to alginate and polyacrylamide, other novel crosslinking strategies have been used to introduce a viscous response into otherwise elastically responding hydrogels. For example, functionalizing PEG with aliphatic hydrazine and aldehyde side groups lead to the formation of reversible hydrazone bonds, yielding a stress-relaxing synthetic hydrogel [68]. Cells encapsulated in hydrogels with stress-relaxing capability were more viable and exhibited a larger projected cell area than cells encapsulated in hydrogels with pure elastic behavior. Lou *et al*. [69] created a natural, stress-relaxing hydrogel by synthesizing an IPN of collagen and hyaluronic acid modified with reversible hydrazone bonds (Fig. 4C). hMSCs encapsulated in these IPN hydrogels spread and induced the formation of focal adhesions via collagen fiber realignment and integrin clustering. Other novel chemistries to introduce viscoelasticity into elastically responding hydrogels have also emerged as this subdiscipline continues to expand rapidly [70–72].

In summary, incorporating viscoelastic properties into ECM-mimicking biomaterials is critical if the local viscoelastic microenvironment of the cell is to be effectively recapitulated. Furthermore, we hypothesize that the complex response of ECs to stiffness in 3D microenvironments might be partially explained by differences in the viscoelastic properties of the materials employed. Further studies are needed to elucidate these mechanisms and thereby gain greater control over the formation of microvasculature in biomaterials.

## Matrix degradability

The degradation of the surrounding ECM is necessary for the formation of healthy blood vessels. Proteolytic enzymes must: (i) degrade the basement membrane to allow sprouting from the main vessel; (ii) degrade ECM material in the vicinity of the sprouting site to allow for EC invasion; (iii) continue the degradation process to create a cavity for the developing lumen [73]. Matrix metalloproteinases (MMPs) are a specific class of proteolytic enzymes that degrade structural proteins such as collagen, release sequestered growth factors from the surrounding matrix, expose integrin sites and detach pericytes from sprouting blood vessels [74, 75]. In particular, MT1-MMP, MMP2 and MMP9 have been identified as essential mediators of sprouting angiogenesis [22, 76, 77]. To better simulate the vascular niche and encourage tissue-level integration, natural polymers or MMP-sensitive cleavage sites have been incorporated into ECM-mimicking biomaterials. For example, the peptides GGPQG↓IWGQK(Dde)AhxC and GCRDGPQG↓IWGQDRCG have been added to PEG and hyaluronic acid hydrogels to track MMP activity [78] and to encourage ECFCs to undergo vasculogenesis *in vitro* [79]. Many of these peptides are well-studied and have a wealth of evidence to support their incorporation; however, in recent years, a new question has been posed: does *relative* material degradability influence differentiation and subsequent endothelial development?

One of the first studies to examine the impact of relative matrix degradability on the behavior of encapsulated cells was conducted by Khetan *et al*. [80]. Briefly, a maleimide group was added to methacrylated hyaluronic acid to create two hydrogels: one that degraded rapidly and another that formed additional crosslinks under UV exposure. The photopolymerized hydrogels were most resistant to degradation and could bias MSC osteogenic/adipogenic differentiation independently of the value of the elastic modulus, thereby indicating that relative matrix degradability is an essential attribute in designing ECM-mimicking biomaterials. In a separate study, PEG hydrogels with oligo(lactic acid) and acryloyl were photopolymerized, nanopatterned and then exposed to a constant concentration of hydrochloric acid over varying time scales to create a range of differentially degraded polymers [81]. MSCs were more viable but spread less on more degraded polymers (i.e. polymers exposed to hydrochloric acid over longer time scales).

**Figure 5.**
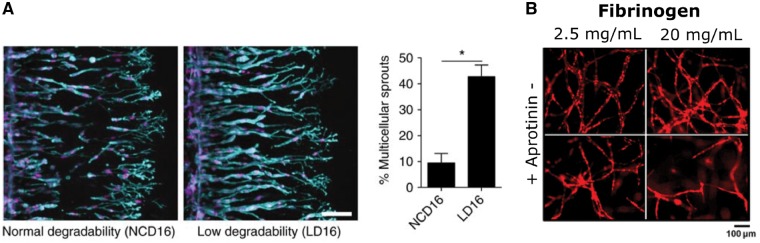
The degradability of the ECM regulates vascular morphogenesis. (A) Decreasing the degradability of methacrylated dextran hydrogels by reducing their susceptibility to MMP-mediated degradation increased the number of multicellular sprouts (cyan highlights F-actin expression). Stiffness remained constant at ∼1 kPa. Reprinted from [83] with permission. (B) Aprotinin, a small molecule commonly used to stabilize fibrin hydrogels, slowed degradation and impaired the ability of mCherry-HUVECs to form vascular networks. Reprinted with permission from [84]

Critically, studies on relative matrix degradability have found applications in microvascular regeneration—most of these studies utilized natural polymers (e.g. collagen, fibrin, hyaluronic acid) that were modified via a novel crosslinking strategy. For example, it was determined that tuning the degradability of collagen hydrogels by introducing EDC/NHS-mediated crosslinks affected matrix-embedded EC gene expression and SMC proliferation *in vitro* as well as the re-reendothelialization of a murine carotid wire injury model *in vivo* [82]. In a pioneering study, Trappmann *et al*. [83] varied the susceptibility of crosslinker sequences to MMP-mediated degradation by introducing an amino acid mismatch in a peptide sequence that is otherwise amenable to degradation (Fig. 5A). Expectedly, EC migration speed was reduced by about 50% in the less degradable hydrogels; more strikingly, less-degradable hydrogels encouraged approximately four times more multicellular sprouts than the more-degradable hydrogels. To tune the formation of microvasculature in fibrin hydrogels, the effect of aprotinin, a commonly employed fibrinolysis inhibitor, was examined [84] (Fig. 5B). Inhibiting the degradation of fibrin hydrogels impaired vascular network formation; however, if aprotinin was removed entirely from the formulation, the fibrin hydrogel became unstable and hindered vessel development.

The studies discussed in this section carefully tuned hydrogel chemistries to ensure a consistent elastic modulus across all experimental conditions at the time of cell seeding. However, as hydrogels degrade, other physical parameters also fluctuate. For example, hydrogel degradation results in the loss of bioactive polymer, which decreases ligand availability and possibly affects the viscoelastic properties of the hydrogel. Further studies are needed to monitor the temporal mechanics of differentially degrading hydrogels.

## Cell-adhesion peptides

In the previous four sections, we primarily focused on the fibrous architecture and force-conduction capabilities of ECM fibrils. Here, we will review the importance of integrin-binding motifs in biomaterials and their effect on EC morphology and vascular network formation. In brief, integrins are membrane-bound heterodimers that serve as the critical bridge between the ECM and the cytoplasm [85, 86]. As such, integrins are vital for cell-to-matrix and cell-to-cell adhesion. To promote integrin-mediated cell anchorage and signaling, the short peptide Arg-Gly-Asp (RGD), derived from both fibronectin and vitronectin, has been widely incorporated into protease-sensitive, synthetic hydrogels. For example, RGD conjugated to heparin-PEG hydrogels promoted the formation of a well-organized cytoskeleton in HUVECs and increased their viability upon encapsulation [87]. RGD-modified PEG hydrogels guided angiogenic sprouting from embedded chick aortic ring explants; substituting aspartic acid (D) with glutamic acid (E) within the RGD sequence completely abrogated sprouting in the hydrogel, confirming the specificity of the peptide’s action [88]. The geometrical organization and concentration of RGD are critical in regulating EC morphogenesis; intermediate concentrations of RGD (20 μg/ml) promoted tubulogenesis, while higher concentrations of the peptide inhibited endothelial vascular assembly [89]. Linear RGDs (targeting β_3_ integrins) immobilized in PEG hydrogels led to increased EC focal adhesion formation when compared to cyclic RGDs (targeting both $\beta_{1}$ and $\beta_{3}$ integrins) [90].

Tyr-Ile-Gly-Ser-Arg (YIGSR) is another short adhesive peptide derived from laminin fragments that has been shown to regulate EC morphology in synthetic and natural matrices. PEG-YIGSR enhanced vasculogenesis in hydrogel constructs cultured *in vitro* and in a murine corneal angiogenesis model; interestingly, the vasculogenic potential of this platform was maximized when YIGSR was combined with RGD peptides, pointing to a possible synergy between the two peptide motifs [91]. The importance of laminin fragments on the regulation of EC morphogenesis was further underscored when it was discovered that the addition of laminin to collagen hydrogels resulted in end-to-end aggregation of ECs and increased VEGF uptake by ECs. This finding is especially significant because the binding sequences contained in collagen, in the absence of high levels of VEGF, failed to initialize tubulogenesis and instead guided ECs to undergo a cobblestone-like morphology [92].

**Figure 6.**
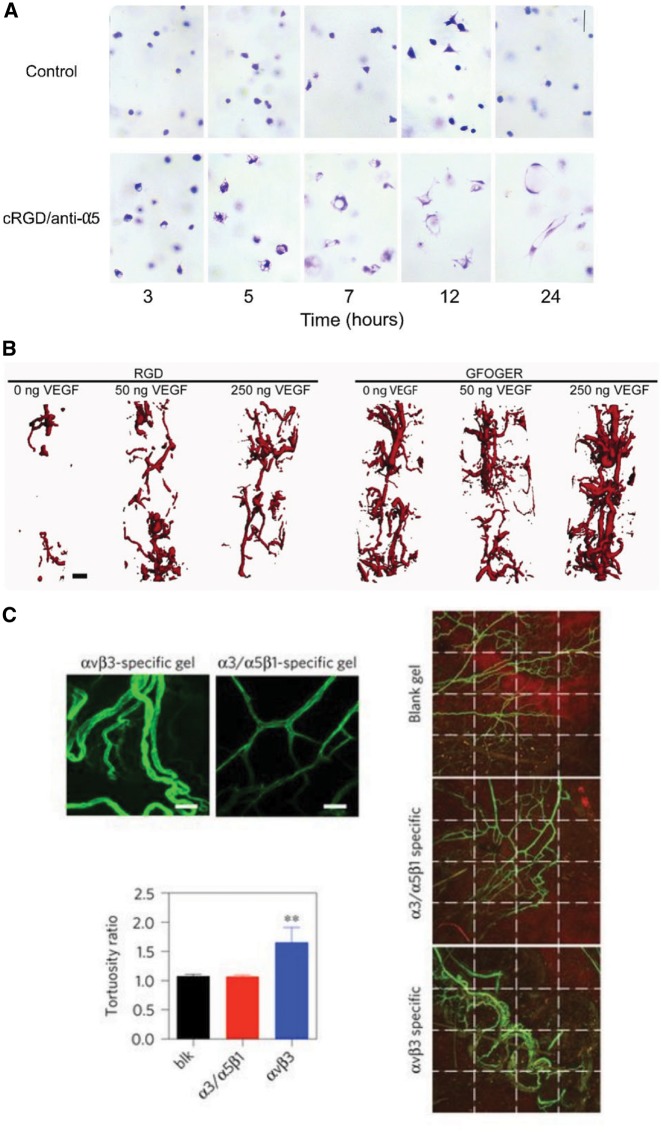
Controlling cell-integrin interactions regulates EC morphogenesis. (A) cRGD peptide and anti-α5 antibodies block the initiation of vasculogenesis in fibrin hydrogels. Reprinted from [94] with permission from Elsevier. (B) GFOGER-modified PEG hydrogels implanted in a radial bone defect showed significantly increased vascularization when compared to RGD-modified PEG hydrogels, even in the absence of growth factors. Reprinted from [96] with permission from John Wiley and Sons. (C) $\alpha_{3}/\alpha_{5}\beta_{1}$- and $\alpha_{v}\beta_{3}$-specific hyaluronic acid-based matrices implanted in a murine Matrigel plug assay differentially regulate the topology of Isolectin-AF488 labeled neovessels. Reprinted by permission from Springer Nature [99]

Recently, greater focus has been placed on restricting which integrins participate in vascular tube formation by carefully controlling the integrin-binding sequences available to encapsulated cells. Vailhé *et al*. [93] suggested that the balance of cell traction forces and the density of the surrounding fibrin hydrogel regulates $\alpha_{V}\beta_{3}$ integrin distribution, which, in turn, could promote the transduction of an angiogenic signal; in addition, $\alpha_{V}\beta_{3}$ integrin distribution was found to be highly dependent on the composition of the surrounding ECM. Dr George E. Davis’ group was among the first to show that $\alpha_{V}\beta_{3}\;$and $\alpha_{5}\beta_{1}$ integrins are critical in regulating tubulogenesis in fibrin hydrogels [94] (Fig. 6A). These integrins are also responsible for controlling EC invasion in response to sphingosine-1-phosphate in fibrin matrices; however, in collagen hydrogels, $\alpha_{2}\beta_{1}$was determined to be the mediator of EC invasion [95]. García *et al*. [96] utilized an $\alpha_{2}\beta_{1}$-specific peptide (GFOGER) to demonstrate that $\alpha_{2}\beta_{1}$ specificity was vital to the revascularization of bone constructs and could encourage blood vessel formation in the absence of VEGF; the integrin $\alpha_{V}\beta_{3}$, in contrast, required supplemental, exogenous VEGF to achieve similar regeneration (Fig. 6B). Therefore, there is a strong possibility that the addition of angiogenic GFs may be partially circumvented by the careful presentation of specific integrin-binding sequences. Though it has been established for more than two decades that the $\alpha_{4}\beta_{1}\;$integrin is present in ECs and binds the Arg-Glu-Asp-Val (REDV) fibronectin motif [97], only recently REDV-functionalized hydrogels were reported to support angiogenesis in otherwise inert alginate scaffolds [98]. Li *et al*. further highlighted the need for integrin-specificity in angiogenic biomaterials (Fig. 6C). By introducing short recombinant vitronectin fragments into hyaluronic acid hydrogels, it was observed that $\alpha_{3}/\alpha_{5}\beta_{1}$ integrins were necessary for the formation of space-filling, mature vasculature; in contrast, the activation of $\alpha_{v}\beta_{3}$ tended to encourage the establishment of a clumped, leaky network [99].

In summary, peptides that promote EC attachment and mobility have long been utilized to guide vascular morphogenesis; recently, the focus has shifted to encouraging specific integrin interactions to form nontortuous, well-branched, mature vasculature. Still, 89% of the published biomaterial studies between 1970 and 2018 used RGD as their motif of choice (recently reviewed by Huettner *et al*. [100]). Given the wealth of available cell-adhesion peptides (CAPs) and the accumulated knowledge about their effect on EC behavior, there is a critical need to develop innovative methods to probe these mostly unexplored CAPs *in vitro* and translate these findings into the clinic.

## Conclusions and future directions

The field of tissue engineering and regenerative medicine has made significant progress in the development of biomimetic, angiogenic biomaterials by utilizing biophysical cues to regulate microvascular morphogenesis. In this review, we have focused on the modulation of ECM density, stiffness, viscoelasticity, degradation and CAPs in both synthetic and natural hydrogels. While the exact mechanism needs further elucidation, it appears that decreasing ECM density leads to greater EC motility and the creation of more branched, extensive vascular networks. The effect of ECM stiffness on vascular morphogenesis is less evident than the impact of varying ECM density, at least in 3D microenvironments. Separate studies that used innovative approaches to decouple ECM density from stiffness reach differing conclusions on the effect of ECM stiffness on sprouting angiogenesis from EC-covered microcarriers [42, 45]. Recent work has given more support to the hypothesis that a biomaterial’s stiffness is closely intertwined with their viscoelastic response and degradation rate. The application of new integrin-binding motifs has led to the creation of innovative biomaterials that demonstrate revascularization in animal models as the scientific community increasingly recognizes the current over-reliance on RGD motifs [96, 99, 100]. From the ongoing discussion, it is evident that a new wave of angiogenic biomaterials presenting physical cues has emerged to aid in microvascular recovery. These materials have the potential to overcome the current limitations of angiogenic protein delivery and cell therapy.

We envision the following directions in developing the next generation of angiogenic, ECM-mimicking biomaterials that will maximize both our fundamental understanding of the vascular milieu and clinical promise. First, ECM-mimicking biomaterials should incorporate physiological viscoelastic properties to ensure that cells can remodel and respond to the tissue-like matrix mechanics. Second, an unexplored plethora of adhesion peptides exists beyond RGD that can be embedded into angiogenic biomaterials as motifs that target specific integrins. Third, the potential of differentiating iPSCs into ECs and perivascular cells must be harnessed to create patient-specific therapies [2, 101–103]. The incorporation of angiogenic biomaterials in these stem cell-based vascular therapies will be critical to avoid off-target differentiation and the formation of malignant teratomas [104]. Fourth, 3D bioprinting has emerged as a technology that has the potential to revolutionize tissue engineering [105, 106]. Significant effort has been devoted to developing biocompatible, printable bioinks that are suitable for supporting the formation of microvasculature; consequently, several candidates have been identified in recent years. GelMA, a known bioink, was recently shown to support tubulogenesis of iPSC-derived ECs [107]. Additionally, collagen and fibrin were found to be the most suitable bioinks for the bioprinting of HUVECs [108]. However, final printing resolution and therefore the ability to 3D print cellular microvasculature remains a significant challenge. Three main bioprinting modalities (extrusion-based, droplet-based and laser-based bioprinting) have been developed to address these challenges, but all three methods remain in need of further refinement before vascularized tissue can be readily fabricated [109]. Fifth, and most importantly, it is critical that angiogenic materials be extensively tested in large animal models to ensure that these revolutionary advances make their way to the clinic and thereby achieve their goal: improving the vascular health of patients.
